# Novel Composite Membranes Based on Chitosan Copolymers with Polyacrylonitrile and Polystyrene: Physicochemical Properties and Application for Pervaporation Dehydration of Tetrahydrofuran

**DOI:** 10.3390/membranes9030038

**Published:** 2019-03-07

**Authors:** Ksenia V. Otvagina, Anastasia V. Penkova, Maria E. Dmitrenko, Anna I. Kuzminova, Tatyana S. Sazanova, Andrey V. Vorotyntsev, Ilya V. Vorotyntsev

**Affiliations:** 1 Laboratory of Membrane and Catalytic Processes, Nanotechnology and Biotechnology Department, Nizhny Novgorod State Technical University n.a. R.E. Alekseev, 24 Minin St., Nizhny Novgorod 603950, Russia; yarymova.tatyana@yandex.ru (T.S.S.); an.vorotyntsev@gmail.com (A.V.V.); ilyavorotyntsev@gmail.com (I.V.V.); 2 St. Petersburg State University, 7/9 Universitetskaya nab., St. Petersburg 199034, Russia; a.penkova@spbu.ru (A.V.P.); m.dmitrienko@spbu.ru (M.E.D.); ai.kuzminova@mail.ru (A.I.K.)

**Keywords:** chitosan, copolymers, composite membrane, selective layer, dehydration, pervaporation, tetrahydrofuran

## Abstract

Pervaporation has been applied for tetrahydrofuran (THF) dehydration with novel composite membranes advanced by a thin selective layer composed of chitosan (CS) modified by copolymerization with vinyl monomers, acrylonitrile (AN) and styrene, in order to improve the chemical and mechanical stability of CS-based membranes. Composite membranes were developed by depositing a thin selective layer composed of CS copolymers onto a commercially-available porous support based on aromatic polysulfonamide (UPM-20^®^). The topography and morphology of the obtained materials were studied by atomic force microscopy (AFM), scanning electron microscopy (SEM) and X-ray diffraction analysis (XRD). Thermal properties and stability were determined by coupled evolved gas analysis (EGA-MS). Transport properties were estimated in pervaporation dehydration of THF. The effect of operating parameters for the pervaporation dehydration of THF such as feed compositions and temperatures (295, 308 and 323 K) was evaluated. It was shown that CS modification with different vinyl monomers led to a difference in physical and transport properties. The composite membrane with the thin selective layer based on CS-PAN copolymer demonstrated optimal transport properties and exhibited the highest water content in the permeate with a reasonably high permeation flux.

## 1. Introduction

Among all membrane processes, pervaporation as a method to separate liquid mixtures was commercialized only in the 1980s. However, it has rapidly developed since that time [1,2]. Now, pervaporation is successfully used for dehydrating organic media [3,4,5,6] and for water desalination [7,8]. The purification and dehydration of conventional solvents such as tetrahydrofuran (THF) is an essential task due to a high demand for this highly volatile substance in chemical processes [9,10,11,12]. THF is widely used as a reaction medium in organic synthesis, a solvent for polymers, an extracting agent for a number of natural compounds and an anti-crystallization additive to rocket and aviation fuels, and it is applied in the production of urethane elastomers and synthetic lubricating oils [11]. It is known that THF tends to generate an unstable hydroperoxide on contact with air. This makes THF dehydration a hazardous process, wherein the concentration of peroxides increases the risk of explosion during distillation [13,14]. Moreover, the separation of the THF-water azeotrope mixture (94.3 wt. % of THF) requires several steps of distillation and additional chemicals which increase the final cost of the solvent [15,16]. These disadvantages could be easily eliminated by using the membrane process, i.e., pervaporation. Pervaporation dehydration of THF allows energy effective one-step separation of the azeotrope composition even under ambient temperatures [17,18]. However, the performance of this process dramatically depends on the type and properties of the membrane material [18]. Therefore, modern membrane technology emphasizes the development of new, efficient, cheap and environmentally friendly pervaporation membranes.

Hydrophilic polymers such as polysaccharides [19,20,21] and polyvinyl alcohol (PVA) [22,23] are commonly used as a membrane material for pervaporation dehydration. Among all hydrophilic polymers, chitosan (CS), a biopolymer obtained from chitin, is now of interest for membrane development due to its antifungal properties, biodegradability (at certain conditions, which allow environmentally safe utilization), high water selectivity, film-forming properties and impressive thermal stability [24,25,26].

Remarkable CS film-forming properties make it possible to obtain both dense and composite membranes. However, since the permeation flux is determined largely by the membrane thickness, the application of composite membranes is preferable. This makes it possible to increase the process performance by depositing a thin selective polymer layer onto a porous support that provides mechanical strength and does not affect mass transfer [27,28]. CS was extensively studied for composite membrane formation [29,30,31,32,33,34]. A CS/Polytetrafluoroethylene composite membrane was used to separate the methanol/toluene mixture by pervaporation [29]. The membrane exhibited a separation factor of 58.4 and permeation flux of 0.13 kg/(m^2^ h) for the separation of azeotrope feed composition (68 wt. % of methanol). The composite CS-polyvinyl alcohol blend membrane on a polyacrylonitrile support was tested for pervaporation dehydration of ethanol–water mixtures [30]. In this case, a CS-polyvinyl alcohol blend was cross-linked with glutaraldehyde in order to obtain stability in presence of a water selective layer. Another CS membrane with a porous poly(ether-block-amide) support was used for the dehydration of n-methyl-2-pyrolidone [31]. For the feed mixture containing 4.6 wt. % water, the membrane exhibited a separation factor of 182 and a water flux of 0.024 kg/(m^2^ h). A noteworthy approach to the design composite membrane for pervaporation processes by combining CS with graphene oxide was recently demonstrated [32,33]. The membrane was tested for Biodiesel production by pervaporation-assisted esterification [30] and methanol dehydration [33]. The selective layer was highly compatible with the support due to ionic complexation between negatively-charged carboxylate ions of graphene oxide and positively-charged protonated amines of CS [33].

CS-based membranes were implemented in THF dehydration by pervaporation [35,36,37]. Krishna Rao et al. showed that cross-linked CS-PVA blend membranes effectively separate a THF-water (5 wt. % of water) mixture by pervaporation, with a separation factor of 4203 and a permeation flux of 0.098 kg/(m^2^ h) at 30 °C [36]. Anjali Devi and colleagues demonstrated a similar approach [37]. Pervaporation membranes based on CS cross-linked with poly(vinylpyrrolidone) (PVP) exhibited a separation factor of 1025 and a water flux of 0.0995 kg/(m^2^ h) for the pervaporation of the azeotrope feed composition (5.69 wt. % of water) at ambient temperatures. In both cases, cross-linking was used to prevent the solubility of CS in water and to improve mechanical stability. The same technique (chemical or thermal cross-linking method) was used to prepare pervaporation CS-based membranes with the desired properties for other separation tasks [38,39,40]. However, sometimes cross-linking leads to a decrease in the selectivity and/or permeability of a membrane [41,42]. Moreover, membrane surface characteristics, mechanical, thermal and transport properties strongly depend on the amount of cross-linking agent in a polymer matrix [37,38,39,40,41,42]. At the same time, the search for a suitable concentration of a cross-linking agent and the optimization of membrane preparation conditions are time- and resource-consuming processes.

In our previous work [43], it was demonstrated that CS could be efficiently modified by radical copolymerization with vinyl monomers; before this, just a few papers were published in this field [44,45]. The main advantage of this modification is that it could be applied with a wide range of monomers, resulting in a notable change in chemical, mechanical and thermal stability, solubility and other essential properties [43,46,47,48]. Radical copolymerization with styrene [47] and acrylonitrile [48] significantly increases CS mechanical properties and, more importantly, limits polysaccharide dissolution in the presence of water without fractional free volume decrease [43].

In the present study, CS copolymers with acrylonitrile and styrene were used for the development of novel pervaporation membranes. A composite structure membrane with a selective layer based on CS was proposed for THF dehydration. A novel approach for the stabilization of a CS-based selective layer was developed in this study—the residual acetic acid that was used for copolymer solution preparation was washed out using a sodium hydroxide solution. The combination of CS copolymerization followed by washing in sodium hydroxide solution is proposed in this work as an alternative method to conventional cross-linking. The transport properties were studied during pervaporation dehydration of THF at various feed compositions and temperatures (295, 308 and 323 K).

## 2. Experimental

### 2.1. Materials

Chitosan (CS, molecular weight (M_W_) = 105 kDA, deacetylation degree (DD) = 80%) was supplied by Bioprogress CJSC (Moscow, Russia). Acrylonitrile (99% pure) was supplied by Lukoil PJSC Subsidiary (Russia) and used after purification by distillation. Styrene (99% pure) and solvents tetrahydrofuran (THF), acetone, dimethylformamide for copolymers synthesis, purification and pervaporation tests were supplied by Sigma-Aldrich (St. Louis, MO, USA) and used after purification by distillation. L-Ascorbic acid (pharmaceutical secondary standard) and hydrogen peroxide solution 30 wt. % in H_2_O were obtained from Himreactive (Nizhny Novgorod, Russia) and used without additional purification. A hydrophilic porous membrane based on aromatic polysulfonamide (UPM-20, pore size 200 Å) was supplied by Vladipor JSC (Russia) and used as the membrane support. THF-H_2_O mixtures for pervaporation were prepared from purified solvents, and the mixture compositions were determined chromatographically.

### 2.2. Copolymers Synthesis

The CS-PAN and CS-PS copolymers were synthesised using a technique proposed by Fedoseeva et al. [49]. The synthesis was conducted in aqueous acetic acid solutions of CS containing 3 wt. % of the polysaccharide in the presence of a redox system composed of ascorbic acid and hydrogen peroxide. Detailed synthesis procedures were described previously [43].

### 2.3. Membrane Preparation

Polymer films (average thickness 40 μm) based on synthesized copolymers were obtained by casting the corresponding solutions onto a polyethylene terephthalate support using an automatic casting knife MemcastTM Plus (POROMETER, Nazareth, Belgium) with a 150 μm blade. Polyethylene terephthalate has a low affinity to CS copolymers and acts as an inert support which easily peels off the membrane after it is dried. The solvent was evaporated in equilibrium conditions: an enclosed area, ambient temperature. Composite membranes with a thin selective layer based on CS copolymers were prepared by casting of the synthesized CS copolymers solutions onto the UPM-20 support surface and desiccation at room temperature to form a selective layer with a thickness of ~2 μm.

In order to provide the CS-based membranes additional water stability, the composite membranes were treated with 40 wt. % NaOH solution and rinsed off with 40 wt. % ethanol solution in water. NaOH solution neutralizes acid traces from the polysaccharide solution, that can promote polymer matrix solubility in the presence of water during the pervaporation process.

### 2.4. Fourier Transforms Infrared Spectroscopy

IR spectra of the samples were recorded using a FTIR spectrophotometer IRAffinity-1 (Shimadzu, Kyoto, Japan). The samples were analysed in KBr matrix and in films. To study the films, an attenuated total reflectance (ATR) accessory with a ZnSe crystal plate (PIKE Technologies, Fitchburg, WI, USA) was used.

### 2.5. Atomic Force Microscopy

The topography of the membranes was evaluated using a scanning probe microscope SPM-9700 (Shimadzu, Japan). The samples were placed on a sample holder using adhesive carbon tabs (SPI Supplies Division of Structure Probe Inc., West Chester, PA, USA) and were cleaned out with ethanol [50,51,52]. The lateral scan area was of up to 30 μm × 30 μm. Images were recorded with a dimension of 256 × 256 pixels in a taping mode using silicon vibrating cantilevers POINTPROBE FMR-20 (Neuchâtel, Switzerland) with a stiffness coefficient of 1.3 N m^−1^ and a typical tip radius of no more than 8 nm and a tip height was from 10 to 15 μm.

According to AFM scanning, an arithmetic average roughness height (R_a_) and a mean roughness depth (R_z_) were obtained. The values were calculated by the SPM Manager ver. 4.02 software (Shimadzu, Japan) based on 512 × 512 matrix.

### 2.6. Scanning Electron Microscopy

The membrane’s morphology (cross-sections) was estimated using a Zeiss Merlin SEM microscope (Zeiss, Oberkirchen, Germany). To study the cross-sections, the membranes were immersed in liquid nitrogen and fractured perpendicular to the surface. The microphotographs were recorded using secondary electrons at a voltage of 1 kV.

### 2.7. X-ray Diffraction Analysis

The membrane’s structure was studied at ambient temperature using D8 DISCOVER diffractometer (Bruker, Billerica, MA, USA) (40 kV, CuKα radiation, step size 0.05°, 40 mA, scan rate 5 s/step) for 2θ = 5–70° in the Bragg-Brentano geometry. The experiments were cared out on the CS, CS-PS, CS-PAN after NaOH treatment. PS and PAN were investigated without NaOH treatment, because they don’t have ionogenic groups and NaOH doesn’t influence their crystallinity.

The d-spacing was calculated from XRD data according to the Bragg’s law. The crystallinity (Cr) of CS and CS copolymers with vinyl monomers was calculated according to the following equation:

Cr % = (I_int_/(I_int_ + I_amorphos_)) × 100% (1)

where I_int_ and I_amorphos_ are the intensities of crystalline and amorphous peaks respectively.

### 2.8. Coupled Evolved Gas-Analysis

It was previously demonstrated that direct EGA-MS effectively complements the characterization of polymeric materials [53]. For direct EGA-MS measurements, a temperature-programmable Multi-Shot Pyrolyzer EGA/PY-3030D (Frontier Laboratories, Fukushima, Japan) was used. Approximately 50 μg of the sample was treated in helium flow 50 ml/min according to the following temperature program: the first stage—exposition at 323 K for 10 min, the second stage—sample heating from 323 K to 1073 K (10 K/min). Identification of the resulting chromatographic peaks was carried out during the second stage of heating by a mass-selective detector GCMS QP-2010 Plus (Shimadzu, Japan). Ionization in the MS was obtained by electron impact (EI) at 70 eV and a mass range between 12 and 500 amu was scanned at a rate of 2000 s/scan. Reaction products were separated on a 2.5 m Ultra ALLOY EGA Tube (Frontier Lab, Japan) at 373 K (45 min) implementing ion monitoring in helium 7.0 (99.99999%) flow. Reaction products were identified with the help of NIST-11 database of mass spectra and “GCMS Real Time Analysis” software.

### 2.9. Pervaporation Experiment

Pervaporation experiments were performed using a laboratory cell with a membrane area of 9.6 cm^2^ under stirring of the feed mixtures at a range of temperatures (295, 308, 323 K) and vacuum mode of pervaporation at a residual pressure below the membrane was less than 0.1 mmHg. The pervaporation set-up consisted of a thermostatically-controlled membrane module (cell), a trap cooling by liquid nitrogen for collecting permeate, a pressure gauge for pressure monitoring at the permeate side of the cell and a vacuum pump. The content of permeate was determined by gas chromatography using a chromatograph “Chromatec-Crystal” with a thermal conductivity detector on a packed column filled with a “PoraPak R1” sorbent. Each pervaporation experiment was conducted at least three times. The mean accuracy for the transport parameters was as follows: for water content in the permeate 2%, for permeation flux 5%.

Permeation flux (*J*, kg/(m^2^ h)) was calculated as,

$$J=\frac{w}{A\times t}$$

where *w* is the mass of water in permeate (kg), *A*—the membrane area (m^2^), *t*—time (h).

Separation factor was calculated as

$$\beta =\frac{y\left(1-x\right)}{x\left(1-y\right)}$$

where *y* is water mass fraction in permeate and *x* is water mass fraction in feed.

To evaluate total pervaporation performance, pervaporation separation index (*PSI*) was calculated as

PSI=J×(β−1)

## 3. Results and Discussion

The influence of CS modification by copolymerization with acrylonitrile and styrene on the polymer structure and physicochemical properties were studied by FTIR spectroscopy, AFM, SEM, XRD, EGA and the evaluation of membranes transport properties was carried out by pervaporation for dehydration of an industrially important solvent tetrahydrofuran.

### 3.1. Membranes Composition

**Figure 1:**
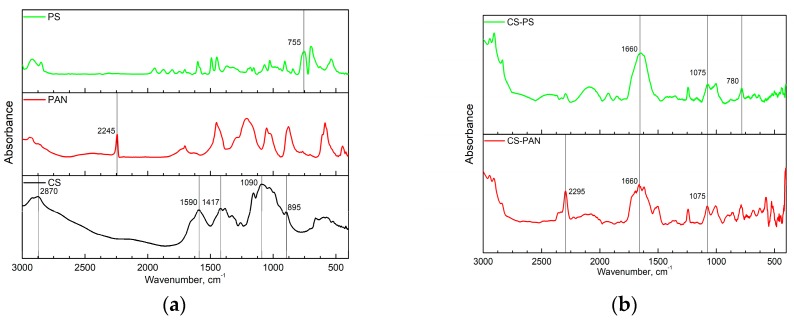
The FTIR spectra of: (**a**) the initial CS, PAN and PS homopolymers, (**b**) CS-PAN and CS-PS copolymers.

Fourier transform infrared (FTIR) spectroscopy analysis of initial CS, PAN and PS homopolymers and the synthesized copolymers was used to characterize membrane composition. FTIR spectra of the copolymers were registered after purification from the corresponding homopolymer on Soxhlet extractor for 48 hours. The obtained FTIR spectra are presented in Figure 1.

The FTIR spectrum in Figure 1a represents the characteristic bands of CS: the CH asymmetric stretching vibration of CH_2_ (2870 cm^−1^); the most important amide II bands—NH stretching (around 1590 cm^−1^) and CH stretching (1417 cm^−1^); asymmetric stretching of a C–O–C bridge (1090 cm^−1^) which is typically observed in the region 1156–1030 cm^−1^; the C–N fingerprint (895 cm^−1^).

Characteristic bands indicating asymmetric stretching of C–O–C bridge in CS appear after copolymerization as well (Figure 1b); however, they shift from 1090 cm^−1^ to 1075 cm^−1^. The intensity of this line in copolymers decreases due to the reaction mechanism—macroradical is generated on the polysaccharide chain through oxidative degradation of the glycosidic bond [49]. Additionally, typical amide I band at 1660 cm^−1^ became more pronounced in copolymers. PAN characteristic band (C≡N stretching at 2245 cm^−1^) appears in copolymer and shifts to 2295 cm^−1^. Stretching vibrations of the C-Ar in substituted benzene ring at 755 cm^−1^ in PS IR spectra also shift in the CS-PS copolymer spectra to the higher wavenumbers (780 cm^−1^). The appearance of PAN and PS characteristic bands confirm the corresponding copolymers formation.

### 3.2. Membranes Morphology

To establish the relationship between the membranes structure and transport properties, the obtained composite membranes with thin selective layer based on CS copolymers were studied by microscopy methods and X-ray diffraction (XRD).

**Figure 2:**
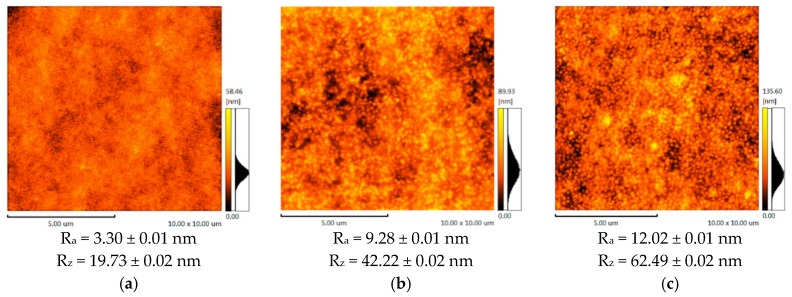
The AFM images and roughness parameters of: (**a**) the support UPM-20, (**b**) CS-PAN/UPM-20, (**c**) CS-PS/UPM-20.

The membranes topography was studied by the atomic-force microscopy (AFM). Figure 2 represents AFM images and roughness parameters (R_a_, R_z_) obtained for the support UPM-20 and the composite membrane surfaces.

**Figure 3:**
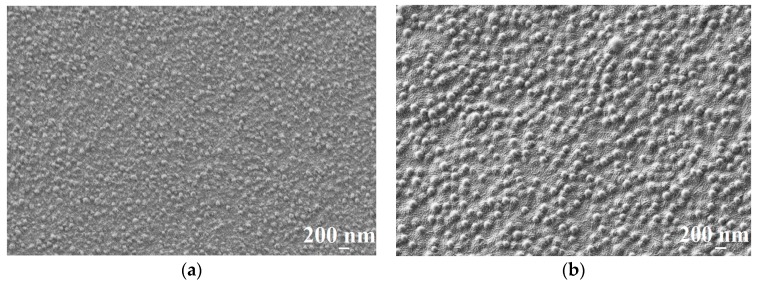
The SEM surface micrographs of (**a**) CS-PAN/UPM-20 and (**b**) CS-PS/UPM-20 membranes.

**Figure 4:**
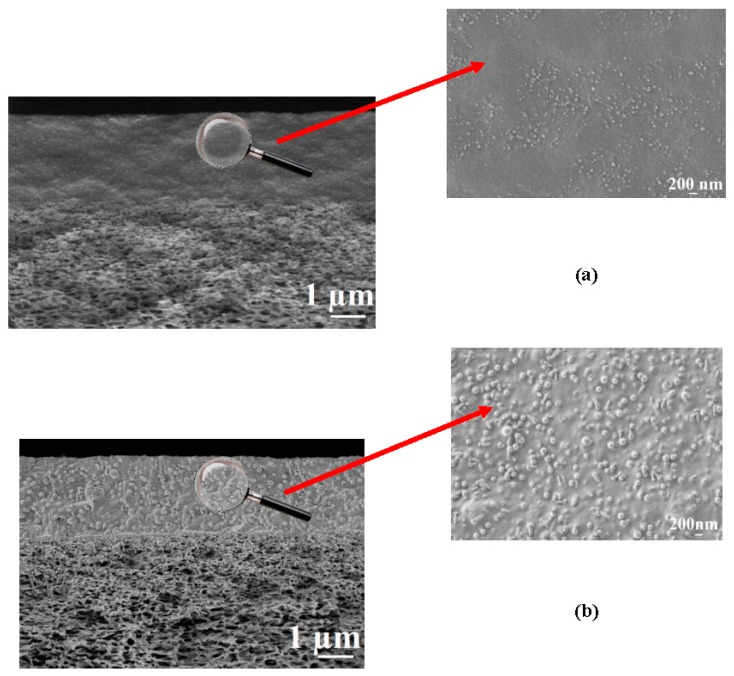
The SEM micrographs of the cross-sections for (**a**) CS-PAN/UPM-20 and (**b**) CS-PS/UPM-20 membranes at different magnifications.

According to the AFM results, the support (UPM-20) large-scale topography (large-scale roughness characterized by R_z_) influences the topography of the obtained composite membranes. The porous support acts as a nucleation center for the thin layer formation, since it is known that substrate topography mediates the topography of the thin films [54,55]. Enlarging the initial support structure leads to at least twice-higher R_z_ in the case of the composite membranes. The CS-PAN and CS-PS have a globular surface structure, which is typical for CS [56] due to the conformational features of polysaccharide macromolecules in a solution. In particular, it was shown that solvated CS forms micelle-like agglomerates (or globular structures). These globules are composed of chitosan units with NH_3_^+^-groups responsible for electrostatic swelling of the globule, resulting in a diameter proportional to CS deacetylation degree [57]. After solvent evaporation, CS surface structure and morphology remain globular. It should be noted that the globular structure is typical only for CS, but not for PS and PAN. The AFM images for the initial CS, PAN and PS were previously reported and discussed [56]. This is also true for CS copolymers with PAN and PS, as could be seen from microscopy results (Figure 2, Figure 3 and Figure 4). Apparently, CS fragments of the copolymer is inside the globules when synthetic fragments are outside. This hypothesis was supported by a wettability test, described in detail previously [43]. The surface of the copolymer films was well wetted by a unipolar liquid, diiodomethane. We suppose that this structural future provides additional stability to the membrane during the dehydration process. The globules’ approximate diameters could be determined from SEM micrographs of the CS-PAN and CS-PS membranes surfaces represented in Figure 3. The approximate diameter of the CS-PAN globules was 50 nm, while in the case of CS-PS, the globules were more pronounced and had an approximate diameter of 120 nm. This could be due to the presence of a bulky substituent, i.e., an aromatic ring, in the synthetic part of the copolymer. The contribution of the globular structure to the total membrane relief is illustrated by the roughness parameter R_a_ that reflects a small-scale roughness (Figure 2). R_a_ growth in case of composite membranes in comparison to initial support is due to the larger diameter of copolymers globules compared to the diameters of the UPM-20 pores.

The composite membrane cross-sections at different magnifications obtained by SEM are presented in Figure 4.

The presence of two regions with different morphologies is clearly observed in the SEM micrographs (1µm) of the composite membrane cross-sections (Figure 4); the upper region is a homogeneous thin selective layer based on the CS copolymers; the second is a spongy-like structure of the porous industrial membrane UPM-20. SEM micrographs demonstrate good adhesion of the selective layers to the porous support and the absence of the copolymer penetration into the support pores. Based on the SEM data, the thickness of the selective layer, in the case of CS-PAN composite membrane, was ≈ 2.5 ± 0.3 μm, and in the case of CS-PS composite membrane, was about 2.1 ± 0.3 μm. The globular structure of the CS copolymers can be also observed in the SEM cross-sectional micrographs (200 nm) (Figure 4). The inner morphologies of the thin selective layer based on CS-PAN and CS-PS correspond well to the surface features of the same copolymers, and are characterized by the same diameter of the globules (Figure 3).

Thus, the SEM micrographs demonstrate different morphology for CS-PAN- and CS-PS-based membranes. The properties of the selective layer strongly depend on the synthetic modifier bonded with the polysaccharide. The difference in the topography and morphology influenced other physicochemical and transport properties of the CS-based membranes, which are discussed in Section 3.3 and Section 3.4.

**Figure 5:**
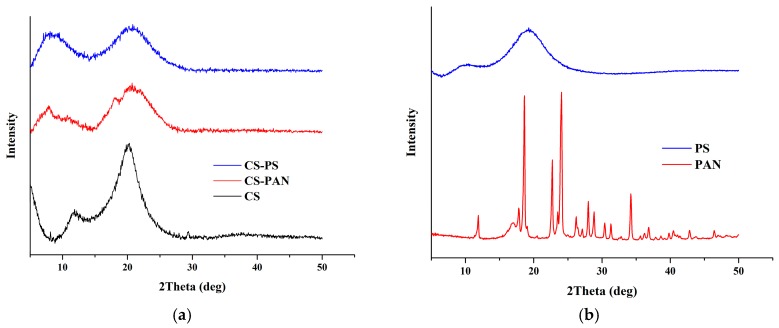
X-ray patterns for (**a**) initial CS, CS-PAN and CS-PS copolymers, (**b**) initial PAN and PS.

X-ray diffraction spectra of initial CS and CS copolymers with PAN and PS are shown in Figure 5. It is known that width and position of the peaks for CS is to a high extent determined by CS deacetylation degree. The XRD pattern of initial CS revealed typical for CS with DD 80% peaks at 2θ of −9° and −17°. The CS XRD data displays a relatively high level of crystallinity (Table 1). However, the XRD patterns for the copolymers demonstrate broader peaks than for initial CS, indicating a lower crystallinity after copolymerization. The CS-PAN crystallinity is much higher than CS-PS crystallinity. The CS crystalline value changes from 79% to 76% in the case of CS-PAN copolymer and to 59% in the case of CS-PS copolymer according to the Equation (1) (Table 1).

The shift of CS peaks and the appearance of new peaks, in the case of the copolymers, indicate successful copolymerization. The d-spacing was calculated according to the Bragg’s law for each peak on the X-ray patterns for initial CS, CS-PAN and CS-PS copolymers and is listed in Table 2. It represents intersegmental distance between polymer chains. The d-spacing was found to shift in the case of CS-PAN from 9.4850 (CS, 2θ = 9.32°) to 10.3738 (CS-PAN, 2θ = 8.52°) and from 5.1384 (CS, 2θ = 17.25°) to 4.9948 (CS-PAN, 2θ = 17.25), and in the case of CS-PS to 13.4682 (CS-PS, 2θ = 6.56°) and 5.0370 (CS-PS, 2θ = 17.60°), where 2θ approximately 9° represent amino- and 2θ approximately 17° represent hydroxyl functional groups of CS which are responsible for intermolecular interactions. During copolymerization, atactic conformation of the PS fragments formed, which results in lower crystallinity of CS-PS copolymer due to irregular structure in comparison to initial CS and CS-PAN copolymer.

### 3.3. Thermal Stability

**Figure 6:**
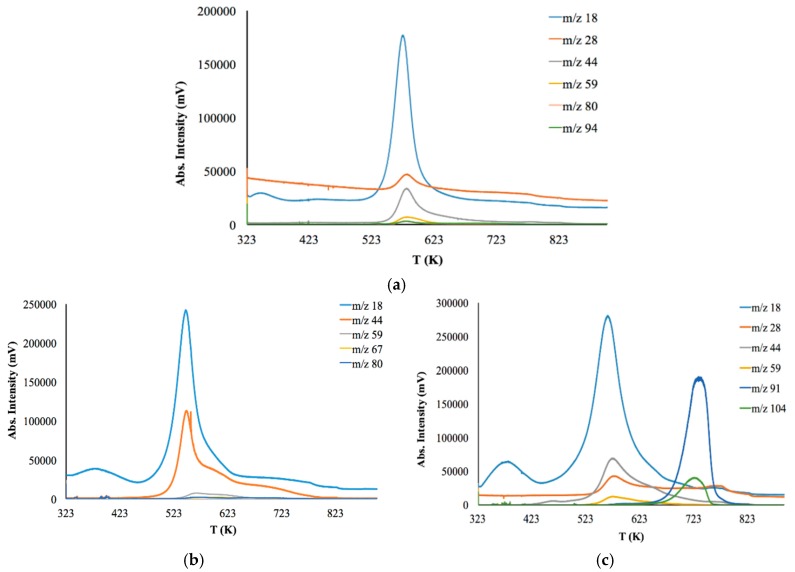
Coupled EGA-MS thermal degradation profiles of the: (**a**) initial CS powder (**b**) CS-PAN copolymer (**c**) CS-PS copolymer.

Coupled Evolved Gas Analysis (EGA-MS) was performed in order to evaluate thermal the stability of initial CS, CS-PAN and CS-PS copolymers. The results are presented in Figure 6 and Table 3. Figure 6 shows the thermal degradation profiles of the samples observed by EGA-MS using a selected ion monitoring (SIM) mode. Here, the SIM curves at different m/z are mainly represented as an asymmetrical peak.

Thermal degradation of initial CS powder involves the following stages (Figure 6a). The first stage is up to 363 K for the evaporation of bounded water in the sample due to the hydrophilic nature of the polysaccharide. The second stage is from 543 to 823 K—deacetylation and depolymerization of CS. It should be noted that copolymers have a lower decomposition temperature than initial CS (523 K). Major degradation of CS-PAN (Figure 6b) occurs at temperatures between 443 and 673 K. This could be due to PAN impact, for which decomposition starts at 333–553 K. Different behavior is observed for the copolymer with the styrene (Figure 6c). The thermal degradation profile for this copolymer has two degradation areas: 493–693 K and 693–763 K. Superficially, the first stage of degradation should be due to PS decomposition, which typically occurs at around 533 K [58]. However, the results of the evolved gases m/z interpretation presented in Table 3 show that during the first stage of degradation, the gas products typical for CS decomposition appear, while styrene is detected only at 653 K.

As previously stated, the nature of a synthetic modifier has a dramatic influence, not only in copolymer morphological features, but also in a range of primary properties, such as thermal behavior and degradation temperature. The results of thermal degradation analysis show that the membranes based on the synthesized copolymers can be operated up to 440 K.

### 3.4. Transport Properties of Composite CS-PAN and CS-PS Membranes

Prospective industrial applications require membrane stability in a wide concentration range of feed mixtures, the possibility to perform a separation process with the membrane at different temperatures, because industrial processes are usually carried out at elevated temperatures, and also the possibility to create a composite membrane with a thin selective layer in order to increase the permeation flux of the membrane. Composite membranes with a thin selective layer based on the synthesized CS-PAN and CS-PS copolymers deposited onto the industrial porous membrane UPM-20 were studied to separate the THF-water mixture by pervaporation and the stability of the obtained membranes was evaluated at elevated temperatures.

As already mentioned in the introduction, tetrahydrofuran (THF) is a widely used organic solvent in-demand for various industries. Dehydration of THF by conventional separation methods (for example, by distillation) is complicated due to azeotrope formation with water (5.7 wt. % water, T_b_ = 338 K) and the presence of hydrogen bonding. The use of pervaporation as a method for dehydration is promising because it allows efficient separation of this mixture without the use of additional reagents and solvents, and has advantages such as low energy consumption, environmental safety and the simplicity of automation and management.

**Figure 7:**
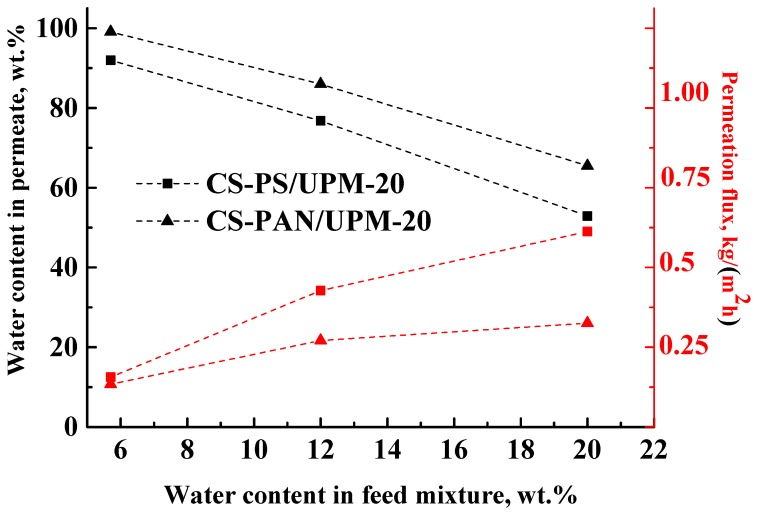
The dependence of water content in permeate and permeation flux on water content in the feed in pervaporation of THF-water mixture by composite membranes based on CS-PAN and CS-PS copolymers at 295 К.

The results of the THF-water mixture (5.7–20 wt. % water) separation during pervaporation by composite membranes based on CS-PAN and CS-PS copolymers at room temperature (295 К) are presented in Table 4 and in Figure 7.

The data in Figure 7 demonstrate that during the separation of all THF-water (5.7–20 wt. % water) feed compositions with the obtained composite membranes, a permeate was enriched with water, which demonstrates the highly selective properties of membranes. However, the water content in the permeate decreased inversely to the water content in the feed for both types of membranes, which could be due to the changes in selective layer morphology caused by intensive CS swelling during separation of more dilute THF solutions. In addition, with the increase of water content (active swelling agent) in the feed, the permeation flux of the penetrants through the membranes increased (Figure 7). These results are due to the fact that chitosan-based membranes are hydrophilic, and water is the active agent for membrane swelling. With the rise of water content in the feed, the swelling of the membranes increased, which led to an increase of the number of transport channels. As in pervaporation, the mass transfer through the non-porous membranes occurs due to the free volume according to solubility diffusion mechanism. With an increase of water content in the feed, the membrane swelling increases, which leads to an increase of the permeation flux and to a decrease of the membrane selectivity (water content in the permeate).

For the CS-PAN membrane, a lower permeation flux and a higher water content in the permeate were observed in comparison to the CS-PS based membrane that could be caused by several factors: (i) a higher intermolecular interaction due to the presence of the polar nitrile groups, more uniform morphology and smaller globules diameter of the copolymer (confirmed by SEM and AFM methods (Figure 2, Figure 3 and Figure 4)), (ii) higher density that led to increased selectivity (confirmed by density measurements with the use of flotation method, Supplementary Materials Section S1.1. Density measurement), and (iii) higher specific area and total pore volume that led to the increased permeation flux (confirmed by porosity measurements, Supplementary Materials Section S1.2. Porometry).

#### 3.4.1. The Influence of Temperature on the Transport Characteristics of Membranes

**Figure 8:**
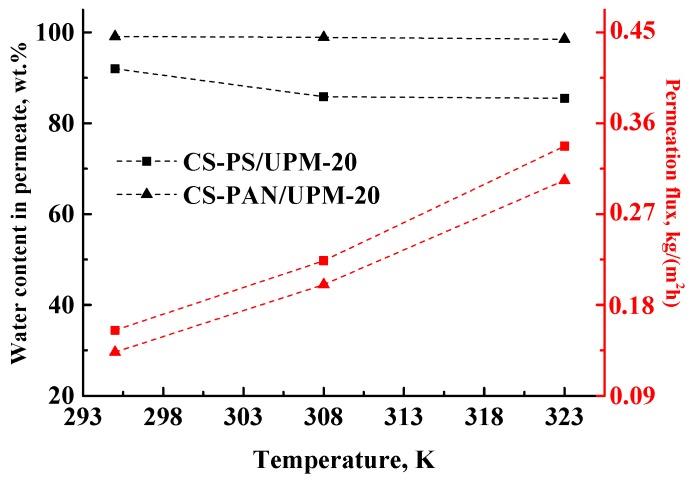
The dependence of water content in permeate and permeation flux on the temperature (295, 308 and 323 K) in pervaporation of an azeotrope THF-water mixture (5.7 wt. % water) by composite CS-PAN and CS-PS membranes.

Most industrial dehydration processes are carried out at elevated temperatures (from 313 K to 473 K [59,60]) to accelerate the separation. Thus, the next goal of this work was to confirm the stability and investigate the transport parameters of the obtained composite membranes at elevated temperatures. The transport properties of the membranes were studied during separation of azeotrope THF-water mixture (5.7 wt. % of water) by pervaporation at 295, 308 and 323 K and presented in Table 5 and in Figure 8. The maximum temperature of the experiment was 323 K, since the boiling point of THF is 338.5 K and the boiling point of azeotropic THF/water (94.3/5.7 wt. %) mixture is 336.9 K at atmospheric pressure [61]. The higher temperatures will lead to the evaporation of components of the feed.

The permeation flux for the obtained composite membranes increases with the increase of temperature. The water content in permeate decreases for the CS-PS membrane from 92 to 85.5 wt. %, while for the CS-PAN membrane it remains almost constant (99.1–98.5 wt. %). Moreover, the membranes remain highly selective with respect to water, even at elevated temperatures (up to 323 K). Thus, CS-PAN composite membrane demonstrated the optimal transport properties for the separation of azeotrope THF-water mixture and high stability at elevated temperatures.

**Figure 9:**
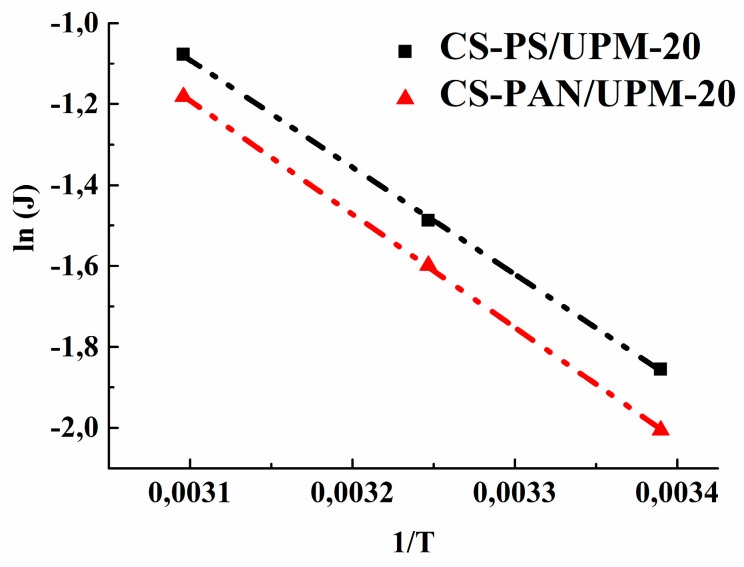
The dependence of ln(J) on 1/T in pervaporation of a THF-water mixture (5.7 wt. % water) by CS-PAN and CS-PS composite membranes.

Based on the obtained data of the permeation flux the activation energy (E_a_) was calculated using the Arrhenius equation for the obtained composite membranes to evaluate the mass transfer process. Figure 9 demonstrates the linear dependence of ln (J) on 1/T, which indicates a change in the permeation flux of obtained membranes according to the Arrhenius equation. The calculated values of E_a_ for CS-PAN and CS-PS composite membranes are presented in Table 6.

The data in Table 6 show that the activation energy for the CS-PS composite membrane is smaller than the value for the CS-PAN membrane, which indicates a lower activation barrier for the mass transfer of low-molecular substances penetrating through the CS-PS membrane. The calculated data are in agreement with the previously evaluated transport properties of the membranes.

#### 3.4.2. Performance Comparison

The pervaporation performances of CS-based membranes in pervaporation dehydration of THF reported in literature and the results of this work are represented in Table 7. It should be mentioned that despite great interest in CS-based membranes for pervaporation dehydration processes, only a few studies are dedicated to the THF-water mixtures separation. Unfortunately, differences in pervaporation conditions (water content in feed, temperature and pressure) don’t make it possible to fully compare the pervaporation performance of the previously reported and obtained in this work membranes. As expected, composite structure with thin selective layer (~2 µm) exhibit elevated permeation flux in comparison to the thick dense polymeric membranes [28,37]. Relatively high values of flux, as a rule, cause lower separation factor.

Thus, the obtained results support the previously reported data. CS is a good choice for membrane design in the case of THF dehydration. CS-based membranes provide relatively high values of pervaporation flux combined with reasonable separation factor. CS thermal stability makes it possible to perform pervaporation processes at elevated temperatures, which promotes mass transfer trough the membrane.

#### 3.4.3. The CS-PAN and CS-PS Thin Selective Layer Stability after Pervaporation of THF-Water Mixtures

**Figure 10:**
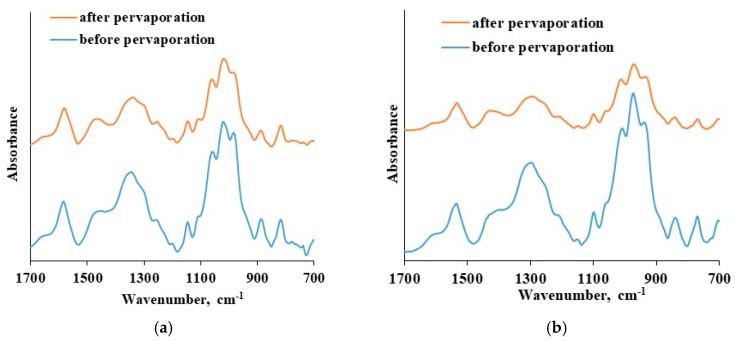
The ATR FTIR spectra of: (**a**) CS-PAN thin selective layer before and after pervaporation, (**b**) CS-PS thin selective layer before and after pervaporation.

The thin selective layer integrity was verified before and after pervaporation tests that were carried out up to 20 wt. % water in the feed at 295 K using ATR-FTIR spectroscopy. The results are presented in Figure 10.

The ATR-FTIR spectra of the CS-based thin selective layer are identical before and after the pervaporation process bough in the cases of CS-PAN and CS-PS. There is an absence of new bands as well as shifts in spectra after pervaporation. This fact indicates the chemical and physical stability of the obtained thin selective layer based on CS copolymers and the preservation of membrane properties when up to 20 wt. % water in the feed applied. Thus, CS copolymerization with vinyl monomers combined with NaOH treatment enables desired membrane design without crosslinking-stage, that could negatively affect the transport properties of the material. Taking into account the study of transport properties of the developed composite membranes based on CS copolymers under different conditions (carrying out pervaporation varying the feed composition and temperatures), it can be concluded that the composite membrane based on CS-PAN copolymer exhibited the best transport properties for the dehydration of THF by pervaporation compared to CS-PS membrane, and that it could be recommended for industrial applications in dehydration processes.

## 4. Conclusions

Novel CS-based membranes with composite structure were developed based on commercially-available aromatic polysulfonamide porous support (UPM-20^®^) and CS copolymers with PAN and PS as a thin selective layer. Copolymerization with acrylonitrile and styrene enabled us to obtain chemically and mechanically stable membranes and to avoid the cross-linking stage, that can lead to a decrease in selectivity and/or permeability of a membrane. The results of thermal degradation analysis demonstrated that the developed membranes based on the synthesized CS copolymers can be operated up to the 440 K.

The effect of operating parameters for the pervaporation dehydration of THF such as feed compositions and temperatures (295, 308 and 323 K) were evaluated. The CS-PAN membrane possessed a lower permeation flux and a higher water content in the permeate in comparison to the CS-PS based membrane, which indicated a higher intermolecular interaction due to the presence of the polar nitrile groups and was associated with a more uniform morphology and smaller globules diameter of the copolymer confirmed by SEM and AFM methods. The stability of the selective layer was verified by ART-FTIR spectroscopy after pervaporation in comparison to resulted obtained before pervaporation.

It was found that the thermal and transport properties of the membranes are dependent on thin selective layer topography, morphology and crystallinity; in other words, on the nature of the CS modifier.

## Figures and Tables

**Table 1:** Chemical structures and abbreviations of the substances, composing copolymers.

Abbreviations	CS	PAN	PS
Chemical structures	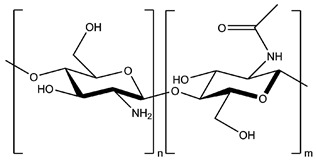	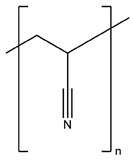	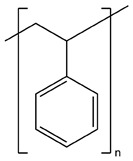

**Table 2:** Values of d-spacing and crystallinity calculated from the XRD data.

Sample	2θ (degree)	d-Spacing, Å	Crystallinity, %
CS	9.32	9.4850	79%
	17.25	5.1384	
CS-PAN	5.16	17.1188	76%
	8.52	10.3738	
	15.29	5.7924	
	17.75	4.9948	
	19.35	4.5852	
CS-PS	5.00	17.6662	59%
	6.56	13.4682	
	17.60	5.0370	

**Table 3:** The main products formed in the temperature degradation of the: initial CS powder, CS-PAN copolymer and CS-PS copolymer.

Sample	m/z	Compound Name	Temperature Range, K	Degradation Temperature, K
initial CS	18	Water	323–363	>523
			523–723	
	28	Nitrogen	543–623	
	44	Carbon dioxide	523–623	
	59	Acetamide	553–613	
	80	Butanedinitrile	563–603	
	94	Dimethylpropanedinitrile	563–593	
CS-PAN copolymer	18	Water	333–443	>443
			443–823	
	44	Carbon dioxide	473–823	
	59	Acetamide	523–663	
	67	Allyl cyanide	553–673	
	80	Butanedinitrile	553–673	
CS-PS copolymer	18	Water	323–443	>443
			443–823	
	28	Nitrogen	523–623	
	44	Carbon dioxide	523–823	
	59	Acetamide	543–673	>543
	91	Methylenecyclohexane	643–823	
	104	Styrene	653–753	

**Table 4:** Pervaporation results for THF-water mixtures at 295 K.

Membrane Composition	Water in Feed (wt. %)	Permeation Flux, kg/(m^2^ h)	Separation Factor (β)	Water Content in Permeate (wt. %)
CS-PS/UPM-20	5.7	0.157	190	92.0
	12	0.428	24	76.8
	20	0.613	4	52.9
CS-PAN/UPM-20	5.7	0.135	1821	99.1
	12	0.271	45	86.0
	20	0.326	7	65.5

**Table 5:** Pervaporation results for THF-water mixture (5.7% water) at different temperatures.

Membrane Composition	Temperature, K	Permeation Flux, kg/(m^2^ h)	Separation Factor (β)	Water Content in Permeate (wt. %)
CS-PS/UPM-20	295	0.157	190	92.0
	308	0.226	100	85.9
	323	0.341	97	85.5
CS-PAN/UPM-20	295	0.135	1821	99.1
	308	0.202	1487	98.9
	323	0.307	1086	98.5

**Table 6:** Activation energy for composite membranes.

Composite Membranes	Е_а_, kJ/mol
CS-PAN/UPM-20	23.3
CS-PS/UPM-20	22.0

**Table 7:** Comparison of pervaporation performance of CS-based membranes in THF pervaporation dehydration process.

Membrane Composition	Thickness, µm	Temperature, K	Feed Composition	J, kg/(m^2^ h)	β	PSI	Reference
Copolymer CS-PS/UPM-20	2	308	5.7 wt. % water	0.226	100	22.4	This work
Copolymer CS-PAN/UPM-20	2	308	5.7 wt. % water	0.202	1487	300.2	This work
Crosslinked blend PVP *-CS	40	308	4.92 wt. % water	0.099	1025	101.4	[37]
Crosslinked blend PVA *-CS (20 wt. % PVA)	35–40	303	5 wt. % water	0.098	4203	411.8	[36]
Mixed matrix CS-NaY Zeolite (40 wt. % NaY)	40	303	5 wt. % water	0.168	2140	359.4	[35]

* PVP—poly(vinylpyrrolidone), PVA—poly(vinyl alcohol).

## Supplementary Materials

The following are available online at https://www.mdpi.com/2077-0375/9/3/38/s1.
