# Supplementary material for: Novel Composite Membranes Based on Chitosan Copolymers with Polyacrylonitrile and Polystyrene: Physicochemical Properties and Application for Pervaporation Dehydration of Tetrahydrofuran
Source: Membranes (Basel). 2019 Mar 7;9(3):38. doi: 10.3390/membranes9030038 (PMC6468362; doi:10.3390/membranes9030038)
Supplement: Supplementary file 1 [file membranes-09-00038-s001.pdf]

Supplementary materials

To prove the reproducibility of the results the porosity and density measurements for the CS-PAN and CS-PS composite membranes on UPM-20 support were carried out.

For this purpose, three parallel membrane syntheses were carried out. The obtained membranes were named as follows: CS-PAN/UPM-20 (1) and CS-PS/UPM-20 (1),CS-PAN/UPM-20 (2) and CS-PS/UPM-20 (2), and CS-PAN/UPM-20 (3), and CS-PS/UPM-20 (3), where number means synthesis number (membrane series).

1.1. Density measurement

The densities of the membranes were determined using the “flotation” method. The method was conducted as follows: a graduated cylinder (25 cm^3) with ground joint and the plug was filed to a half with a mixture of isopropanol and carbon tetrachloride in the volume ratio 1:1. Isopropanol (ρ=0,79 g/cm3 @ 20°C) and carbon tetrachloride (ρ=1,60 g/cm3 @ 20°C) were chosen because they don’t react with the measured samples and don’t cause their swelling and solvation. Then a piece of a membrane sample was put down into the cylinder. According to the position of the membrane sample in the liquid, more of a low density or a high-density liquid was added and mixed with the glass mixer in the way that a membrane sample takes an equilibrium position in the very middle of the liquid column according to the graduation. Then a density of the obtained mixture of liquids was measured using a pycnometer (10 cm^3). Density was measured three times on each membrane. The results of the measurements are represented in Table S1.

Table S1. The results of density measurements.

Membrane	ρ, g/cm^3
CS-PAN/UPM-20 (1)	1.087± 0.001
CS-PAN/UPM-20 (2)	1.087± 0.001
CS-PAN/UPM-20 (3)	1.088± 0.001
CS-PS/UPM-20 (1)	1.041±0.003
CS-PS/UPM-20 (2)	1.040± 0.001
CS-PS/UPM-20 (3)	1.041± 0.001

The data presented in Table S1 demonstrate good reproducibility, the difference lies within the error. CS-PAN/UPM-20 membrane has a higher density in comparison to CS-PS/UPM-20 membrane.

1.2.Porometry

Study of the porous characteristics (specific surface area, pore size distribution and total pore volume) of the obtained membranes was conducted on the Sorbi – MS (Meta Ltd., Russia) with advanced term training of samples. The study of solids based on the interpretation of the IV type

of adsorption isotherms with a hysteresis loop. By interpreting this type of isotherms, it is possible to estimate the specific surface area by BET and STSA with acceptable accuracy, as well as to obtain an approximate characteristic of pore size distribution and full pore volume. The results are presented in Table S2 and Figure S1.

Table S2. The results of porometry.

Membrane	Specific surface area (BET), m^2/g	Specific surface area (STSA), m^2/g	Total pore volume, cm^3/g
CS-PAN/UPM-20 (1)	7.0 ± 0.3	7.1 ± 0.1	0.008
CS-PAN/UPM-20 (2)	6.9 ± 0.3	6.8 ± 0.1	0.007
CS-PAN/UPM-20 (3)	7.1 ± 0.3	7.2 ± 0.1	0.008
CS-PS/UPM-20 (1)	7.5 ± 0.2	7.3 ± 0.2	0.009
CS-PS/UPM-20 (2)	7.7 ± 0.2	7.5 ± 0.2	0.010
CS-PS/UPM-20 (3)	7.4 ± 0.2	7.3 ± 0.2	0.009

	CS-PAN/UPM-20	CS-PS/UPM-20
(1)	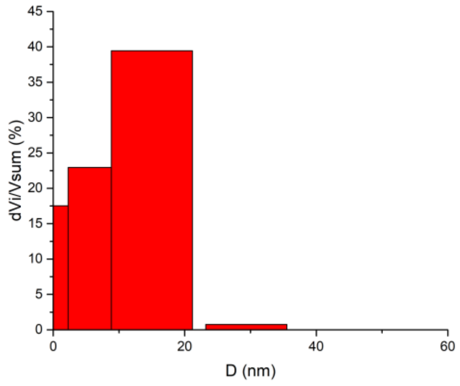	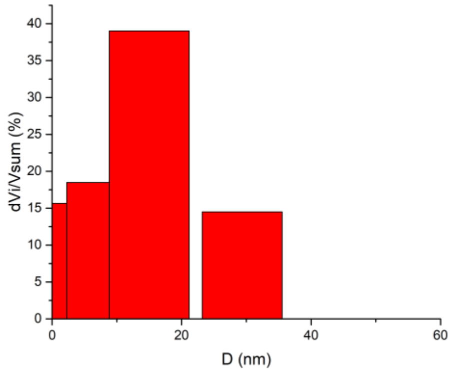
(2)	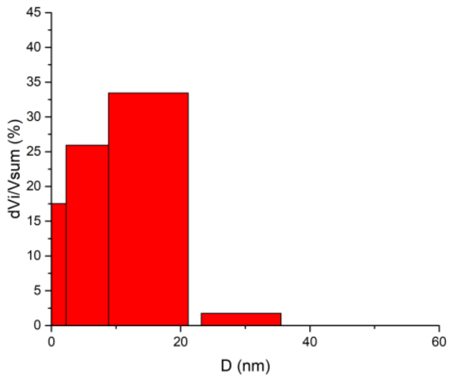	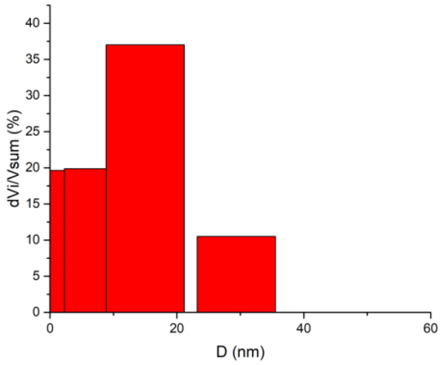

(3)	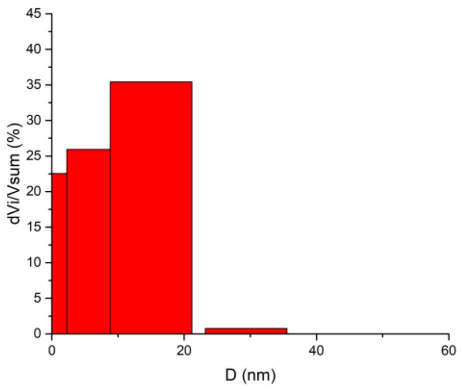	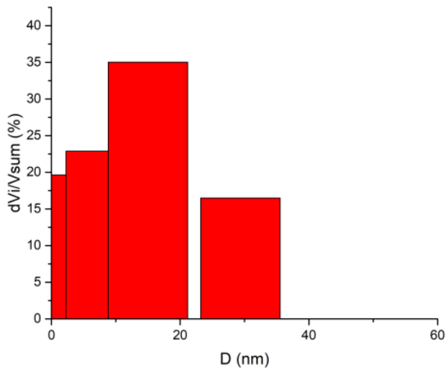

Figure S1. Pore size distribution of relative total volume for three series of CS-PAN/UPM-20 and CS-PS/UPM-20 membranes

Figure S1. represents pore size distribution calculated from the adsorption isotherms. Hear the ratio of the pore volume (dVi) of a certain size (D) to the total pore volume (Vsum) is represented. The results of porometry have good reproducibility. For both CS-PS/UPM-20 and CS-PAN/UPM-20 the results are in the agreement with the support porosity where most of the pores lie around 20±10 nm. For CS-PAN/UPM-20 membrane lower volume of pores with the size of more than 20 nm is observed. Greater flexibility of PAN micro chains in comparison to PS, which has a bulk substitute in a monomer unit.
